# Genetic Variants in Interleukin-10 Gene Association with Susceptibility and Cervical Cancer Development: A Case Control Study

**DOI:** 10.1055/s-0042-1743262

**Published:** 2022-02-25

**Authors:** Pushpendra D. Pratap, Syed Tasleem Raza, Ghazala Zaidi, Shipra Kunwar, Sharique Ahmad, Mark Rector Charles, Ale Eba, Muneshwar Rajput

**Affiliations:** 1 Central Research Laboratory, Molecular Diagnostic Unit, Department of Biochemistry, ERA's Lucknow Medical College, Era University, Lucknow, Uttar Pradesh, India; 2 Department of Allied Health Sciences, Era University, Lucknow, Uttar Pradesh, India; 3 Department of Obstetrics & Gynecology, Era University, Lucknow, Uttar Pradesh, India; 4 Department of Pathology, Era's Lucknow Medical College, Era University, Lucknow, Uttar Pradesh, India

**Keywords:** human papillomavirus, cervical cancer, gene polymorphism

## Abstract

**Objectives** Cervical cancer (CC) is one of the most destructive disease caused by persistent HPV infection which affects women worldwide, especially in developing countries. The genetic basis of host immune response especially cytokine function has been shown to influence CC susceptibility. Studies have demonstrated that IL-10 gene polymorphism have been associated with numerous malignancies, but in context to CC results were inconclusive. Though, aim of our study to investigate the association between IL-10 -1082A/G and -819C/T promoter polymorphism and CC susceptibility.

**Material and Methods** This study comprised 192 women with CC and 200 controls. HPV detection was done by RT-PCR and genotyping was assessed through PCR-RFLP method. Serum concentration of IL-10 measured by ELISA.

**Results** Women with AG and AG+GG genotypes of IL-10 -1082A/G had two-fold increased risk of CC [OR, 2.35 (95% CI, 1.54–3.58), *p* = 0.005], [OR, 2.03 (95% CI, 1.36–3.04), *p* = 0.0005] compared to controls. Women with G allele of -1082A/G polymorphism had linked with CC susceptibility [OR, 1.39 (95% CI, 1.02–1.88), *p* = 0.036] compared to controls. No significant difference was found between patients and controls in the genotype or allele frequencies of IL–10 -819C/T polymorphism [OR, 1.00 (95% CI, 0.63–1.58), *p* = 0.99]. The level of serum concentration of IL-10 was significantly higher in cases compared to controls.

**Conclusion** These findings help to understand that polymorphism of IL-10 -1082A/G gene is associated with increased risk of CC development and can serve as a marker of genetic susceptibility to CC.

## Introduction

Cervical cancer (CC) is one of the most fatal malignancies among females and is recognized as the second most common cancer globally. 1 Overall, it is recognized as the fourth most common type of malignancy and frequently occurs in the lower end of the uterine cervix, affecting normal cervix epithelial tissues and causing aberrant changes in the deeper tissues. 2 3 4 CC affects more than half a million women annually, culminating in more than 570,000 diagnosis and 300,000 fatalities worldwide as per WHO reports. Interestingly, 90% of CC patients emerge within low- and middle-income countries. 5 6 There are ∼365.71 million women in India over the age of 15 years who are at risk of CC. According to existing evidence, around 132,000 new cases of CC are diagnosed each year in India, with 74,000 fatalities, accounting for nearly one-third of CC deaths worldwide. Indian women have a 2.5% lifetime risk of CC and a 1.4% lifetime risk of mortality from CC. 7

The human papillomavirus (HPV) is recognized to be the most common cause of CC in women, especially high-risk HPV subtypes HPV16 and HPV18. However, HPV infection is not always essential for the progression of this disease, as the majority of the patients (70–90%) eradicate the virus within 1 to 2 years of initial diagnosis. 8 Besides HPV, various other risk factors for CC development include timing of the first intercourse, early pregnancy, multiple pregnancies in a short period of time, multiple sexual partners, oral contraceptive pills, race or ethnicity, smoking habit, diet habits, parturition, family history of CC, and inadequate socioeconomic circumstances. 9 10 Furthermore significantly, evidence has demonstrated that genetic heredity is one of the most common intrinsic factors associated with the development of CCs, increasing the risk by ∼27%. 11 12 HPV-related epithelial transformation of the cervix, which is a crucial determinant for the development of cervical neoplasia, has been linked to genetic polymorphisms in various immune mediators. 13

Through the activation of various immune components, such as cells and cytokines, the immune system plays a critical role in repressing or encouraging tumorigenesis. The modification of tumorigenesis appears to be associated with the antigen presentation response, which is downregulated in CC, resulting in humoral response and cellular response reduction. 14 Regardless of the fact that HPV infection causes CC in a small percentage of infected women, there is a latent period after infection and before diagnosis of cancer, trying to demonstrate that cell-mediated immunity is a part of regular host immune activity controlled by cytokines, and cytokines' activities are extremely crucial for CC development. 9 Variations in host genetic vulnerability and immunological responses have been linked to increased risk of HPV-related CC. 15

Interleukin-10 (IL-10) is a multifunctional cytokine that is secreted by lymphocytes, monocytes, and other cells and has anti-inflammatory and immunosuppressive characteristics. 16 17 IL-10 is found on human chromosome 1 (1q31–1q32) and has five exons and four introns. 18 Studies have shown that IL-10 is relevant to tumor egregiousness and mediates angiogenesis in tumor tissues. 17 It has been established that IL-10 suppresses activated lymphocytes and macrophages from producing proinflammatory cytokines in these cells. 19 It was reported that rs1800896 (-1082A/G) was found to be related with pediatric postbronchiolitis asthma in a recent study of four single nucleotide polymorphisms (SNPs) in the IL-10 gene, including rs1800871 (-819C/T), 1,800,872 (-592C/A), rs1800890 (-3575 T/A), and rs1800896 (-1082A/G). 20 According to a meta-analysis, rs1800872 may have a role in the development of smoking-related cancer susceptibility. 17

IL-10 genetic variations have been associated with a greater risk of CC throughout many studies. For instance, a recent incident analysis in Chinese women demonstrated that the IL-10 rs1800872 polymorphism is associated with the severity of cervical neoplasia. 21 Another study in the Chinese population explored the correlation between the IL-10 gene polymorphisms rs1800871, rs1800872, and rs1800896, and concluded that rs1800871 potentially contributes to the risk of CC. 22 A meta-analysis in the Asian population, conducted by Ni et al, 23 found a significant correlation between the minor allele of rs1800872 and enhanced cervical carcinogenesis recurrence. However, no association with the rs1800896 polymorphism was observed. rs1800872 has also been associated with persons of Indian, 24 Mexican, 25 and Dutch ethnicities. 26 The genetic polymorphism of IL-10 (-1082A/G) has also been linked to CC in Brazilian 27 and Japanese women. 28

The frequency and intensity of disease occurrences in different regions of the world may differ based on geographical and biological diversity, as well as ethnical differences in genetic makeup. Even within the same ethnicity or origin, differences can be observed. 29 30 Therefore, we conducted the current case control study to determine the association of IL-10 SNPs rs1800870 (-1082A/G) and rs1800871 (-819C/T) polymorphisms with CC susceptibility.

## Material and Methods

### Study Design

#### Patients and Sample Collection

For this case control study, 392 patients were included, of which 192 patients were diagnosed with CC and confirmed by a pathologist based on histopathology and clinical features, and 200 control subjects were negative for cytological abnormalities with no history of cancer and infection, and free from any acute or chronic pathology. All the participants were recruited from the OPD, Department of the Obstetrics and Gynecology, ERA's Lucknow Medical College & Hospital (ELMC&H), Lucknow, Uttar Pradesh. All histopathologically confirmed CC cases were staged as per the International Federation of Gynecology and Obstetrics (FIGO) criteria. Institutional Ethics Committee gave their approval (Ref. No. ELMC &H/2019/R_Cell/EC/169). The purpose and procedures of this study were explained and written informed consent was obtained from all participants.

All study participants underwent DNA analysis for IL-10 polymorphism genotyping. Each subject's peripheral blood was collected into tubes containing ethylenediaminetetraacetic acid (EDTA) and plain vials prior to radiation therapy and/or chemotherapy at the time of initial diagnosis. Each participant's blood and serum samples were kept at –70°C until further use. Structured questionnaire was applied concerning sociodemographic characteristics reproductive as well as sexual behavior and clinical information was obtained from the hospital record section.

#### Genomic DNA Extraction and Genotyping

Genomic DNA from peripheral blood samples of each subjects was extracted using the PureLink Genomic DNA extraction kit according to manufacturer's instructions (Thermo Fisher Scientific). DNA concentration of all samples was measured by NanoDrop 2000c Spectrophotometer (Thermo Fisher Scientific) at 260 nm and purity was assessed through a 260/280 ratio.

#### Enzyme Linked Immunosorbent Assay (ELISA)

**Fig. 1:**
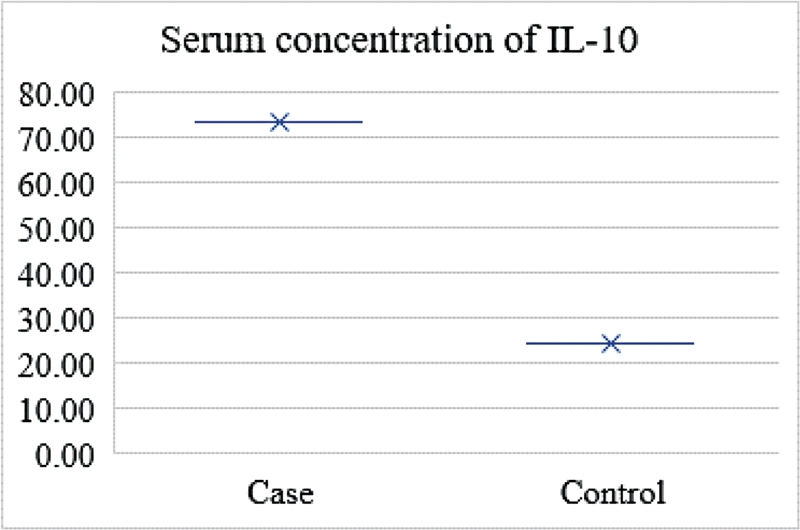
Showing elevated serum concentration of IL-10 in cases versus controls.

The measurement of concentration of IL-10 in serum samples was assessed through enzyme-linked immunosorbent assay (ELISA) using Human IL-10 ELISA Kit (Diaclone, Cat. No.950.060.096) as per the manufacturer protocol ( Fig. 1 ). In brief, 100 μL of samples, controls, and standard were added to the plate and incubated at room temperature for 1 hour. After three washes with washing buffer, 100-μL streptavidin-HRP was added and incubated at room temperature for 30 minutes. The plate was washed three times with washing buffer and 100-μL substrate solution was added for color development. After adding 100-μL stop solution, optical density (OD) was measured and the results were recorded in picograms per milliliter (pg/mL).

#### HPV Detection

For HPV DNA extraction, tissue biopsy samples were collected in normal saline and extracted through HPV 16/18 RT-PCR kit (Liferiver, Shanghai) as per manufacturer protocol. DNA extraction buffer was supplied in the kit, which was thoroughly thawed and spun down in the centrifuge. The tissue was crushed, mixed with 1 mL normal saline (NS), vortexed vigorously, and centrifuged at 13,000 rpm for 5 minutes. The supernatant was discarded and the pellet was again mixed with 1 mL NS, and centrifuged at 13,000 rpm for 5 minutes. The supernatant was discarded, 50 μL buffer was added, vortexed for 10 seconds, and incubated at 100°C for 10 minutes. After that, it was centrifuged for 5 minutes at 13,000 rpm and the supernatant was collected, that is, the DNA was extracted, which can be used as a template for polymerase chain reaction (PCR).

**Fig. 2:**
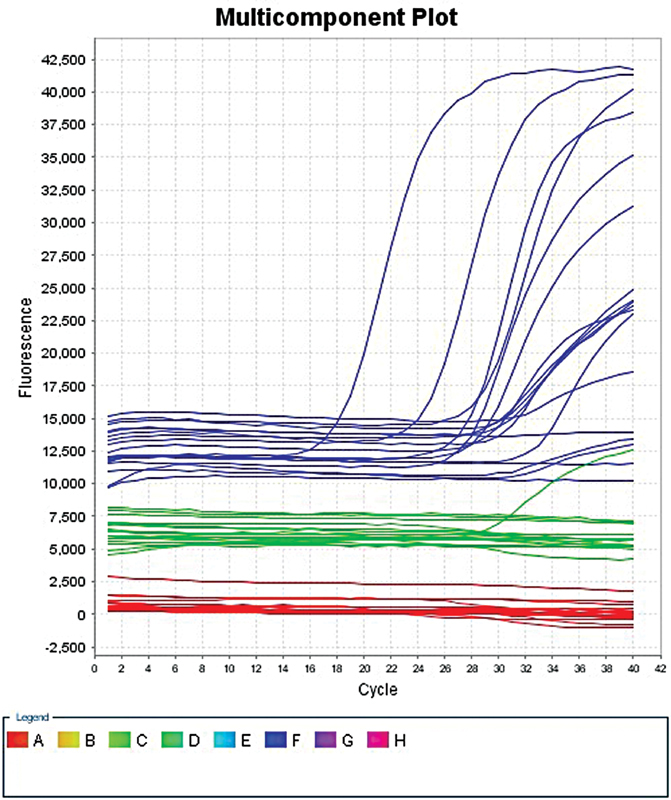
Real-time polymerase chain reaction (PCR) multicomponent graph showing HPV 16 ( *blue line* ), HPV 18 ( *green line* ), and internal control ( *red line* ) expression in cases and control.

Real-time PCR (RT-PCR) was performed in a 40-µL reaction mixture (RM) containing ∼4 µL of extracted DNA (template) and 36 µL of master mix (MM). The MM for each reaction was prepared through pipetting 35 µL of reaction mix (HPV serotype 16 and 18 reactions mix), 0.4 µL of enzyme (DNA polymerase), and then 1 µL of internal control (IC) ending up with a total of 36.4 µL of MM. The RT-PCR cycling conditions included were initial one cycle at 37°C for 2 minutes, then one cycle denaturation at 94°C for 2 minutes, followed by 40 cycles at 93°C for 15 seconds, and at 60°C for 60 seconds. Amplified HPV16 and HPV18 DNA fragment detection was performed in fluorimeter channel FAM and HEX/VIC/JOE with the fluorescent quencher BHQ1 at 60°C ( Fig. 2 ).

#### IL-10 -1082A/G Polymorphism Genotyping

Genomic DNA from peripheral blood samples was used for detection of IL-10 -1082A/G polymorphism by multiplex PCR technique (MJ Mini Thermo Cycler, BioRad). Primers sequence used for IL-10 -1082A/G gene amplification are as follows: the forward primers (5′-TCT GAA GAA GTC CTG ATG TCA CTG-3′) and reverse primers (5′-ACT TTC ATC TTA CCT ATC CCT ACT TCC3′). PCR conditions were 10 pmol of each primer, 2.5 mmol/L MgCl _2_ , 0.2 mmol/L of each dNTPs, 1 unit of *Taq polymerase* (Bioline Ltd., London, UK) along with 100 ng of peripheral genomic DNA with annealing temperature of 55°C.

**Fig. 3:**
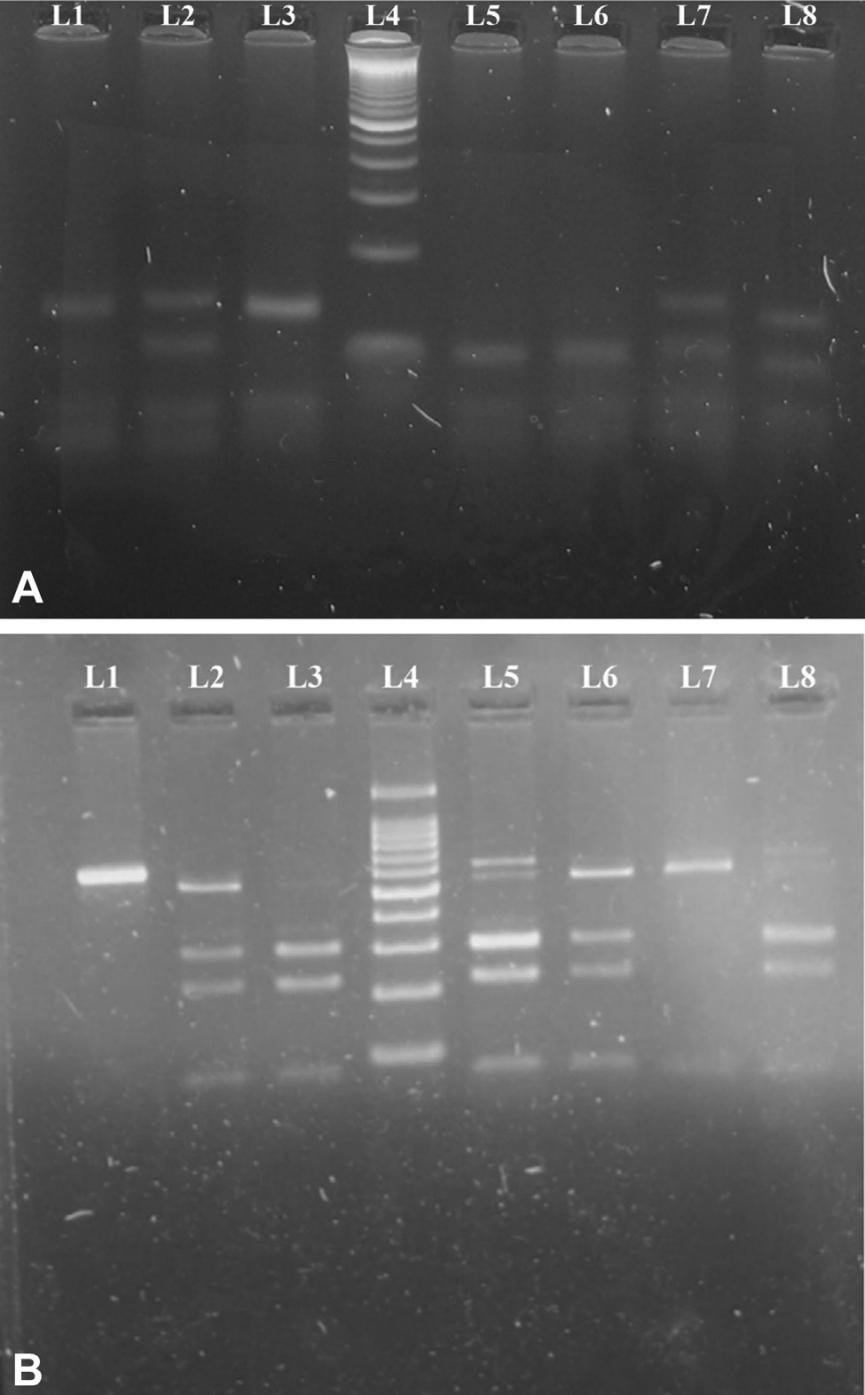
( **A** ) Agarose gel picture showing IL-10 -1082 genotypes lanes 1 and 3: AA genotype (138 bp, 58bp); lanes 2, 7, and 8: AG genotype (138 bp, 110 bp, and 58bp); lane 4: Molecular Marker 100 bp; lanes 5 and 6: GG genotype (110 bp, 58bp). ( **B** ) Agarose gel picture showing IL-10 -819 genotypes. Lane 1: UD (600 bp); lanes 2, 5, and 6: CT genotype (509 bp, 292 bp, 217 bp, 79 bp); lanes 3 and 8: CC genotype (292 bp, 217 bp, 79 bp); lane 4: Molecular Marker 100 bp; lane 7: TT genotype (509 bp, 79 bp).

The IL-10 -1082A/G product amplification corresponds to a 196-bp fragment. The enzymatic restriction was assessed through restricted fragment length polymorphism (RFLP) using amplified product in the presence and using 2 U of the restriction enzymes *MnII* (New England Biolabs, Beverly, Massachusetts, United States) and incubated at 37°C overnight. The products were analyzed by electrophoresis in a 3% agarose gel stained with ethidium bromide (EtBr), and visualized under ultraviolet light. This enzyme cleaves the amplified fragment of DNA in the presence of adenine, producing fragments of 110 and 58 bp, and fragments of 110, 58, and 28 bp in the presence of guanine allele ( Fig. 3A ).

#### IL-10 -819C/T Polymorphism Genotyping

The IL-10 -819C/T polymorphism was performed by PCR followed by RFLP using specific set of primers, forward primer (5′-ATCCAAGACAACACTACTAA-3′) and reverse primer (5′-TAAATATCCTCAAAGTTCC-3′) to amplify a region of 509 bp of IL-10 containing the polymorphic locus. PCR conditions were 0.1 mM of dNTPs, 0.25 uM of each primer, 1.5 mM of MgCl _2_ , 100 ng of DNA, and 1 U of *Taq DNA polymerase* along with an annealing temperature of 57°C. The amplified products were submitted to enzymatic restriction with 2.5 U of *MaeIII* enzyme, at 55°C for 6 hours, for polymorphism analysis. The restriction fragments were electrophoresed on 3% agarose gel stained with EtBr, and visualized under ultraviolet light ( Fig. 3B ).

### Statistical Analysis

All the differences in sociodemographic and categorical data between CC patients and controls were analyzed using contingency tables and chi-squared test ( *χ* 2 test) and the values were expressed along with percentage. Age were tested for normality by Kolmogorov–Smirnov test and a non-normal distribution was assumed. Allele frequency was calculated as [1( *h* + 2 *H* )]/2 *N* , where *h* represents the heterozygous genotype, *H* is the homozygous genotype, and *N* is the sample size for each population. Hardy–Weinberg equilibrium in CC patients and controls was tested using the *χ* ^2^ test. Differences in the genotype frequencies between CC cases and controls were assessed by the *χ* ^2^ test. The SPSS v. 22.0 tool (SPSS Inc., Chicago, Illinois, United States) was used to analyze all the data. The odds ratio (OR) and their 95% confidence intervals (95%CI) from multivariate logistic regression analysis were used to determine the correlations between genotypes and CC risk. A statistically significant *p* value of <0.05 was considered.

## Results

### Sociodemographic Data

For this case control study, 392 women were included and identified as CC patients (192/48.98%) and controls (200/51.02%) according to the histopathological and clinical characteristics. The mean age of the CC patients was 49 ± 11 years, while control samples has a mean age of 44 ± 9 years. Sociodemographic characteristic samples of cases and controls are presented in Table 1 . Information regarding age, parity, educational status, ethnicity, tobacco chewing, and smoking status were compared between the CC and control groups. A higher frequency of parity was observed in women who had a parity of between 3 and 5 ( *p* = 0.0003), were younger than 30 years old ( *p* 0.00001), lived in rural areas ( *p* 0.0001), had lower education ( *p* 0.00001), were smokers ( *p* = 0.001), and had the habit of chewing tobacco ( *p* = 0.0001).

**Table 1:** Sociodemographic characteristics of cervical cancer patients and controls

Controls, *N* = 200			Cases, *N* = 192		*p* -value a	OR	95%CI	*p* -Value
Age (y)	No.	%	No.	%				
21–30	6	3	10	5.2	<0.00001	1.00	Reference	
31–40	62	31	32	16.67		0.31	0.10–0.93	**0.03**
41–50	102	51	76	39.59		0.45	0.16–1.28	**0.12**
51–60	16	8	44	22.92		1.65	0.52–5.28	0.39
≥ 60	30	15.62	14	7		1.29	0.39–4.25	0.67
Parity								
≤2	52	26	30	15.62	**0.0003**	1.00	Reference	
3–5	100	50	134	69.79		2.32	1.38–3.90	**0.001**
≥6	48	24	28	14.59		1.01	0.53–1.93	0.97
Socioeconomic status								
Upper middle	164	82	176	91.67	**0.004**	1.00	Reference	
Lower middle	36	18	16	8.33		0.41	0.22–0.77	**0.004**
Age at marriage								
<18	84	42	64	33.34	0.07	1.00	Reference	
18–30	116	58	128	66.66		1.45	0.96–2.18	0.07
Place of residence								
Urban	74	37	38	19.8	**0.0001**	1.00	Reference	
Rural	126	63	154	80.2		2.38	1.51–3.76	**0.0001**
Religion								
Hindu	96	48	146	76.04	**<0.00001**	1.00	Reference	
Muslim	76	38	44	22.91		0.38	0.24–0.60	**0.0001**
Sikh	28	14	2	1.05		0.05	0.01–0.20	**<0.001**
Educational status								
<5th	60	30	88	45.84	**<0.00001**	1.00	Reference	
5th–8th	44	22	72	37.5		1.12	0.68–1.84	0.66
≥8th	96	48	32	16.66		0.23	0.14–0.38	**<0.0001**
Smoking status								
No	184	92	156	81.25	**0.001**	1.00	Reference	
Yes	16	8	36	18.75		2.65	1.42–4.96	**0.001**
Tobacco chewing status								
No	143	71.5	96	50	**0.0001**	1.00	Reference	
Yes	57	28.5	96	50		2.51	1.65–3.81	**0.0001**

Note: Bolded values are significant.

a Analysis by two-sided chi-squared ( *χ* ^2^ ) test and *p* < 0.05 set as significance level (SPSS Inc., Chicago, IL, United States).

### Histopathological and FIGO Data

Histopathological features with staging as per FIGO criteria and tumor size are presented in Table 2 . Keratinizing squamous cell carcinoma (KSCC) had a higher incident rate (164/85.42%) followed by nonkeratinizing SCC (20/10.42%) and adenocarcinoma (8/4.16%). As per biopsy report, mild dysplasia (20/10%) was more common, followed moderate and severe dysplasia. Stage II cancer was more common in women diagnosed with cervical carcinoma (110/57.30%). A higher frequency was observed in women who had a 2- to 4-cm tumor size (114/59.30%).

**Table 2:** Histopathological and clinical features of cervical cancer patients

Histopathological analysis	No.	%
Nonkeratinizing SCC	20	10.42
Keratinizing SCC	164	85.42
Adenocarcinoma	8	4.16
Dysplasia		
Mild	20	10.5
Moderate	4	2.9
Severe	0	0
FIGO staging		
Stages I	42	21.87
Stages II	110	57.3
Stages III	30	15.62
Stages IV	10	5.21
Size of tumor		
<2cm	38	19.8
2–4 cm	114	59.3
>4 cm	34	17.8
Not specified	6	3.1

Abbreviations: FIGO, International Federation of Gynecology and Obstetrics; SCC, squamous cell carcinoma.

### IL-10 -1082A/G Polymorphism

IL-10 -1082A/G polymorphism genotype distribution among CC patients and controls are shown in Table 3 using the Hardy–Weinberg equilibrium (HWE). Genotype and allele frequency distributions of SNPs among the cases and controls were significantly different ( *p* = 0.0005). The HWE of both controls and CC patients was consistent ( *χ* ^2^ = 2.86, *p* = 0.09 and *χ* ^2^ = 11.9, *p* = 0.0005, respectively). The distribution of genotypes showed no deviation from HWE for both the cases and controls ( *χ* ^2^ = 1.39, *p* = 0.23 and *χ* ^2^ = 1.08, *p* = 0.297, respectively).

**Table 3:** Distribution and association analysis of IL-10 genotypes among cervical cancer cases healthy controls

IL-10 1082 A/G	Genotype frequencies						Association analysis				
	Controls		HWE		Cases		HWE		Cases vs. controls		
Genotype	*N* = 200	(%)	*χ* ^2^	*p* -value	*N* = 192	(%)	*χ* ^2^	*p* -value	OR	95%CI	*p* -value
AA	112	56			74	38.54			1	Reference	
AG	69	34.5	2.86	0.09	107	55.73	11.91	**0.0005**	2.35	1.54–3.58	**0.0006**
GG	19	9.5			11	5.73			0.88	0.39–1.95	0.74
AG + GG	88	44			118	61.46			2.03	1.36–3.04	**0.0005**
Allele											
A	293	73.25			255	66.4			1	Reference	
G	107	26.75			129	33.6			1.39	1.02–1.88	**0.036**
Carriage rate											
A (+)	181	90.5			181	94.27			1	Reference	
A (−)	19	9.5			11	5.73			0.58	0.27–1.25	**0.16**
G (+)	88	44			118	61.46			1	Reference	
G (−)	112	56			74	38.54			0.49	0.33–0.74	**0.005**
IL-10 819 C/T genotype											
CC	63	31.5			61	31.77			1	Reference	
CT	91	45.5	1.39	0.23	88	45.84	1.08	0.297	1	0.63–1.58	0.99
TT	46	23			43	22.39			0.97	0.56–1.66	0.89
CT + TT	137	68.5			131	68.23			0.99	0.65–1.51	0.95
Allele											
C	217	54.25			210	54.69			1	Reference	
T	183	45.75			174	45.31			0.98	0.74–1.30	0.9
Carriage rate											
C (+)	154	77			149	77.6			1	Reference	
C (−)	46	23			43	22.4			0.97	0.60–1.55	0.88
T (+)	137	68.5			131	68.23			1	Reference	
T (– )	63	31.5			61	31.77			1.01	0.66–1.55	0.95

Abbreviation: HWE, Hardy–Weinberg equilibrium.

Overall, three genotypes (A/A, A/G, and G/G) and two alleles were found in this study. The control group had the following genotypes: A/A in 112 (56%) women, A/G in 69 (34.5%) women, and G/G in 19 (9.5%) women. Among the cancer patients, 74 (38.54%) had the A/A genotype, 107 (55.73%) had the A/G genotype, and 11 (5.73%) had the G/G genotype. The allele frequencies were 0.73 for the A allele (ancestral allele) and 0.27 for the G allele (variant allele) in the controls, while among the CC patients allele frequencies were 0.66 and 0.34 for A and G alleles, respectively.

Genotypes and allele frequencies for statistical analysis included the dominant model and carriage rate, and are summarized in Table 3 . Frequency of the A/G genotype was higher in cases as compared with controls ( *p* = 0.0006). A higher frequency of A/G + G/G genotype was observed in CC patients as compared with controls ( *p* = 0.0005). G allele frequency was higher in CC cases ( *p* = 0.036) as compared with controls. Consequently, susceptibility to CC was higher in women carrying the A/G genotype (OR: 2.35; CI95%: 1.54–3.58), A/G + G/G genotypes (OR: 2.03; CI95%: 1.36–3.04), and G allele (OR: 1.39; CI95%: 1.02–1.88). A significant association was found between the IL-10 -1082A/G polymorphism and CC.

### IL-10 -819C/T Polymorphism

IL-10 -819C/T polymorphism genotype distributions among CC cases and controls are summarized in Table 3 . Overall, three genotypes (C/C, C/T, and T/T) and two alleles (C and T) were observed in this study. Genotypes frequencies were 63 (31%) for C/C, 91 (45.50%) for C/T, and 46 (23%) for T/T in the control group, and 61 (31.77%) for C/C, 88 (45.84%) for C/T, and 43 (22.39%) for T/T, respectively, in CC patients. The allele frequencies were 0.54 for allele C (ancestral allele) and 0.46 for T allele (variant allele) in the controls and 0.54 and 0.46 for alleles C and T, respectively, among the CC patients. No significant association was found when compared with the controls ( *p* > 0.05).

An association between IL-10 -1082A/G gene polymorphism in tobacco chewers along with smokers, summarized in Table 4 . In comparison to the controls, there was no association was found among genotype and allele frequencies between tobacco chewers and smokers ( *p* > 0.05). IL-10 -819C/T gene polymorphism analysis revealed that no significant association was found in tobacco chewers and smokers when compared with the controls (p>0.05).

**Table 4:** Distribution of genotypes and allele frequencies in tobacco chewing and smoking patients of cervical cancer and controls

IL-10 1082 A/G	Tobacco chewing with controls vs. cases				Association cases vs. controls			Smoking with controls vs. cases				Association cases vs. controls		
Genotype	*N* = 57	(%)	*N* = 96	(%)	OR	95%CI	*p* -value	*N* = 16	(%)	*N* = 36	(%)	OR	95% CI	*p* -value
AA	23	40.35	39	40.62	1	Reference	Reference	8	50	16	44.45	1	Reference	Reference
AG	28	49.13	53	55.21	1.12	0.56–2.22	0.75	7	43.75	17	47.22	1.21	0.36–4.12	0.75
GG	6	10.52	4	4.17	0.39	0.10–1.54	0.17	1	6.25	3	8.33	1.5	0.13–16.82	0.74
AG + GG	34	59.65	57	59.37	0.99	0.51–1.93	0.97	8	50	20	55.56	1.25	0.38–4.07	0.71
Allele														
A	74	64.91	131	68.223	1	Reference	Reference	23	71.87	49	68.05	1	Reference	Reference
G	40	35.09	61	31.77	0.86	0.53–1.41	0.55	9	28.13	23	31.95	1.2	0.48–3.00	0.69
Carriage rate														
A (+)	51	89.47	92	95.84	1	Reference	Reference	15	93.75	33	91.67	1	Reference	Reference
A (−)	6	10.53	4	4.16	0.37	0.10–1.37	0.12	1	6.25	3	8.33	1.36	0.13–14.21	0.79
G (+)	34	59.65	57	59.37	1	Reference	Reference	8	50	20	55.56	1	Reference	Reference
G (−)	23	40.35	39	40.63	1.01	0.52–1.97	0.97	8	50	16	44.44	0.8	0.25–2.60	0.71
IL-10 819 C/T genotype														
CC	21	36.84	32	33.33	1	Reference	Reference	6	37.5	13	36.11	1	Reference	Reference
CT	25	43.86	44	45.84	1.16	0.55–2.41	0.7	8	50	12	33.34	0.69	0.19–2.59	0.58
TT	11	19.3	20	20.83	1.19	0.48–2.99	0.7	2	12.5	11	33.55	2.54	0.42–15.21	0.29
CT + TT	36	63.16	64	66.67	1.17	0.59–2.32	0.65	10	62.5	23	63.89	1.06	0.31–3.59	0.92
Allele														
C	67	58.77	108	56.25	1	Reference	Reference	20	62.5	38	52.78	1	Reference	Reference
T	47	41.23	84	43.75	1.11	0.69–1.77	0.66	12	37.5	34	47.22	1.49	0.64–3.50	0.35
Carriage rate														
C (+)	46	80.7	76	71.17	1	Reference	Reference	14	87.5	25	69.45	1	Reference	Reference
C (−)	11	19.3	20	20.83	1.1	0.48–2.50	0.81	2	12.5	11	33.55	3.08	0.60–15.92	0.16
T (+)	36	63.16	64	66.67	1	Reference	Reference	10	62.5	23	63.89	1	Reference	Reference
T (−)	21	36.84	32	33.33	0.86	0.43–1.70	0.65	6	37.5	13	36.11	0.94	0.28–3.19	0.92

For histological grade, all 192 patients were included because they had the histological classification on the basis of the biopsy report. Considering the influence of IL-10 1082 A/G polymorphism on lesion development, the dominant model was adopted to make a better distribution among genotypes and significant association ( *p* = 0.0003) was observed ( Table 5 ) while statistical analysis of IL-10 819 C/T polymorphism revealed no significant association between genotype and allele distribution, as shown in Table 5 .

**Table 5:** Genotypic analysis of IL-10 gene polymorphism with types of cervical cancer

IL-10 1082 A/G	Controls	NKSCC	Association analysis			KSCC	Association analysis			Adenocarcinoma	Association analysis		
Genotype	*N* = 200 (%)	*N* = 20 (%)	OR	95%CI	*p* -value	*N* = 164 (%)	OR	95%CI	*p* -value	*N* = 8 (%)	OR	95%CI	*p* -value
AA	112 (56)	9 (45)	1	1	1	62 (37.80)	1	1	1	3 (37.50)	1	1	1
AG	69 (34.50)	8 (40)	1.44	0.53–3.92	**0.46**	95 (57.93)	2.49	1.60–3.86	**0.0003**	4 (50)	2.16	0.47–9.96	0.31
GG	19 (9.50)	3 (15)	1.96	0.49–7.92	**0.33**	7 (4.27)	0.67	0.27–1.67	0.38	1 (12.50)		1.96 (0.19–19.89)	0.56
AG + GG	88 (44)	11 (55)	1.56	0.62–3.92	**0.34**	102 (62.20)	2.09	1.37–3.19	**0.0005**	5 (62.50)	2.12	0.49–9.12	0.3
Allele													
A	293 (73.25)	26 (65)	1	1	1	219 (66.77)	1	1	1	10 (62.50)	1	1	1
G	107 (26.75)	14 (35)	1.47	0.74–2.93	**0.26**	109 (33.23)	1.36	0.99–1.88	**0.05**	6 (37.50)	1.64	0.58–4.63	0.34
**IL-10 819 C/T genotype**													
CC	63 (31.50)	7 (35)	1	1	1	51 (31.09)	1	1	1	3 (37.50)	1	1	1
CT	91 (45.50)	9 (45)	0.89	0.32–2.51	0.82	76 (46.34)	1.03	0.64–1.66	0.89	3 (37.50)	0.69	0.14–3.54	0.65
TT	46 (23)	4 (20)	0.78	0.22–2.83	0.7	37 (22.57)	0.99	0.56–1.76	0.98	2 (25)	0.91	0.15–5.69	0.92
CT + TT	137 (68.50)	13 (65)	0.85	0.33–2.24	0.74	113 (68.90)	1.02	0.65–1.59	0.93	5 (62.50)	0.77	0.18–3.31	0.72
Allele													
C	217 (54.25)	23 (57.50)	1	1	1	178 (54.27)	1	1	1	9 (56.25)	1	1	1
T	183 (45.75)	17 (42.50)	0.88	0.45–1.69	0.69	150 (45.73)	1	0.75–1.34	0.99	7 (43.75)	0.92	0.34–2.52	0.87

There was a significant association between IL-10 1082 A/G polymorphism genotypes and clinical staging of CC patients categorized as per the FIGO criteria ( *p* = 0.0006, 0.0009, and 0.27) as summarized in Table 6 . No association was observed between IL-10 819 C/T genotypes and clinical staging of CC patients ( Table 6 ).

**Table 6:** Association analysis of SNPs (-1082A/G and -819C/T) in the promoter region of IL-10 gene with clinical stage of cervical cancer cases

IL-10 1082 A/G	Controls		CC with I + II		OR	95% CI	*p-* value	CC with III + IV		OR	95% CI	*p* -value
Genotype	*N* = 200	(%)	*N*	(%)				*N*	(%)			
AA	112	56	58	38.16	1.00	Reference	Reference	16	40	1.00	Reference	Reference
AG	69	34.5	88	57.89	2.5	1.57–3.85	**0.0006**	19	47.5	1.9	0.93–4.00	0.07
GG	19	9.5	6	3.95	0.6	0.23–1.61	0.31	5	12.5	1.8	0.60–5.62	**0.27**
AG + GG	88	44	94	61.84	2.1	1.34–3.17	**0.0009**	24	60	1.9	0.96–3.81	**0.06**
Allele												
A	293	73.3	204	67.1	1.00	Reference	Reference	51	63.8	1.00	Reference	Reference
G	107	26.8	100	32.9	1.3	0.97–1.86	0.076	29	36.3	1.6	0.94–2.58	0.08
IL-10 819 C/T genotype												
CC	63	31.5	49	32.24	1.00	Reference	Reference	12	30	1.00	Reference	Reference
CT	91	45.5	73	48.03	1.03	0.64–1.67	0.9	15	37.5	0.87	0.38–1.97	0.73
TT	46	23	30	19.73	0.84	0.46–1.52	0.55	13	32.5	1.48	0.62–3.55	**0.37**
CT + TT	137	68.5	103	67.76	0.97	0.61–1.52	0.88	28	70	1.07	0.51–2.25	0.85
Allele												
C	217	54.3	171	56.25	1.00	Reference	Reference	39	48.8	1.00	Reference	Reference
T	183	45.8	133	43.75	0.92	0.68–1.25	0.59	41	51.3	1.25	0.77–2.02	**0.36**

## Discussion

CC has been associated with a significantly increased rate of cancer-related mortality among women in underdeveloped nations. 31 CC develops and progresses as a result of several factors. Surprisingly, a prominent fundamental cause of cervix malignancies is the host genetic component or genetic variation. 32 Furthermore, it has been established that cytokine production varies widely among individuals and that these disparities may be determined by genetics. Extensive genome-wide association studies (GWAS) or genetic association studies have been conducted to determine the genetic variables that make a woman vulnerable to developing this carcinogenicity. 33 34 35 At least 50 polymorphic loci have been identified thus far, including -2849, -2776, -2769, and -2763 to date. 36 IL-10 -1082A/G (rs1800870), -819T/C (rs1800871), and -592C/A (rs1800872) are the three most prevalent SNPs in the promoter region that have been demonstrated to have a substantial impact on gene transcription and expression. 37 According to certain genetic study, these polymorphisms potentially enhance and/or change an individual's susceptibility to many cancers, especially head and neck cancer, gastric cancer, leukemia, and others. 38 39 40 Our research looked into whether the IL-10 rs1800870 (-1082A/G) and rs1800871 (-819C/T) polymorphisms are relevant to CC, and we established an association between them.

The correlation between numerous genetic variants in distinct cytokine genes has been evaluated, and it was observed that these polymorphisms enhance the risk of CC. Inflammatory mediators seem to be crucial signaling molecules produced by various cells in the human body. They are connected to the innate immunity, which plays a significant role in establishing the immunological response to cancers and viral infections. 41 42 As a consequence, genetic variations in cytokine-related coding genes may have the potential to cause cancer by altering their function or creating an excessive number of cytokines. 43 44 45

IL-10 is a major anti-inflammatory cytokine with anti-angiogenic and immunosuppressive characteristics. Genetic variants in the IL-10 gene have been found to affect cytokine levels reported by various studies. As a consequence, IL-10 could have both tumor-protective and tumor-promoting properties. 46 47 Multiple studies have looked at both enhancement and deterioration in levels of IL-10 in CC. 48 A case control study published by Stanczuk et al revealed that IL-10 -1082A/G gene polymorphism was significantly associated with increased risk of CC and their findings suggested that women carrying the A/G genotype were associated with higher risk of CC in the African population. 49 According to Singhal et al, the A/G genotypes may considerably enhance the risk of CC development when compared with the AA genotype in the IL-10 -1082A/G polymorphism. 24 Our current study shows that IL-10 rs1800870 (-1082A/G) polymorphism is associated with increased CC susceptibility. In this study, women carrying A/G and A/G + G/G genotypes showed 2.35-fold and 2.03 times significantly higher risk ( *p* = 0.0006 and 0.0005) and women with G allele were also significantly associated with increased risk of CC ( *p* = 0.036) as compared with controls. The findings of our study are in accordance with those of various previous ethnic studies. IL-10 -1082G allele was significantly associated with the development of CC among Zimbabwean population as compared with the IL-1082A allele. 24 Matsumoto et al also established an association between IL-10 -1082G allele and CC susceptibility as compared with the IL-1082A allele among Japanese women. 28 According to Chagas et al, the IL-10 -1082 variation locus may play a imperative role in the progression and development of CC in the Brazilian population. 27 Our study demonstrated that women carrying the A/G genotype have higher risk of CC, followed by those carrying the G/G genotype as compared with those carrying the A/A genotype among nonkeratinizing SCC and controls summarized in Table 5 ( *p* = 0.46). In the context of KSCC, there was a higher incident of A/G and A/G + G/G compared with the A/A genotype ( *p* = 0.0003 and 0.0005), while no association was found between adenocarcinoma and IL-10 1082A/G polymorphism. In this study, there was no significant association between IL-10 -819C/T polymorphism and CC with subgroup analysis as compared with controls. A significant association was found between the IL-10 1082A/G genotype and the clinical stage of CC ( *p* = 0.0006 and 0.0009) compared with the controls ( Table 6 ).

## Conclusion

The finding of our study revealed significant association of IL-10 1082A/G polymorphism with CC susceptibility. Women carrying A/G and A/G + G/G genotypes have an increased risk of CC, suggesting an association with the presence of G allele, confirmed by allele analysis. Furthermore, due to the high prevalence and mortality of CC, our results highlighted the need to extend prevention efforts regarding the disease's significance and urge gynecological follow-ups. The development of a susceptibility framework comprising individual characteristics that increase the risk of CC development, as well as genetic biomarkers such as the IL-10 -1082A/G polymorphism, could be a potential method for assessing the risk of CC. Elevation of serum level is also associated with increased risk of CC compared with controls.
